# Promoting Personhood in Family Conversations with Individuals Living with Dementia

**DOI:** 10.1093/geroni/igaf122.2531

**Published:** 2025-12-31

**Authors:** Chizobam Nweke, R Amanda Cooper, Hannah Brocksmith

**Affiliations:** University of Connecticut, Storrs, Connecticut, United States; University of Connecticut, Storrs, Connecticut, United States; University of Connecticut, Farmington, Connecticut, United States

## Abstract

As the population of adults aged 65 and above keeps rising in the US and other countries around the world, so does the burden of dementia, with significant impacts on the quality of life of this population. This study explored the various ways through which family members promote the personhood of their loved ones with dementia. Thirty-one audio-recorded conversations between people with dementia, Mage 76.65 (SD 13.35), and their family members were obtained from the StoryCorps online archive and analyzed. The following were identified as the strategies through which family members affirmed personhood: reminding the people with dementia of their identities, encouraging them to share their thoughts, assessing and adjusting to their conversational needs, and cultivating relational connections with them. This study also found that the task of promoting personhood through family relationships is complex and there is a need to ensure that adequate accommodation is provided, as too much or too little can be counterproductive. Also, the findings from this study provide further evidence that people with dementia can have positive and meaningful experiences while navigating their illness at home among loved ones.

